# Soluble guanylyl cyclase as a therapeutic target in chronic obstructive pulmonary disease (COPD)

**DOI:** 10.1186/2050-6511-14-S1-P25

**Published:** 2013-08-29

**Authors:** Constantinos Glynos, Dimitris Toumpanakis, Zongmin Zhou, Peter Brouckaert, Theodoros Vassilakopoulos, Andreas Papapetropoulos

**Affiliations:** 1 George P Livanos and Marianthi Simou Laboratories, Evangelismos Hospital, 1st Department of Pulmonary and Critical Care, University of Athens, Athens, Greece; 2 Laboratory of Molecular Pharmacology, Department of Pharmacy, University of Patras, Patras, Greece; 3 Department of Biomedical Molecular Biology, Ghent University, Ghent, Belgium; 4 Department of Molecular Biomedical Research, VIB, Ghent, Belgium

## Background

Resistive breathing (RB) due to airflow limitation is the pathophysiologic hallmark of chronic obstructive pulmonary disease (COPD). Nitric oxide (NO) is a physiological regulator of smooth muscle tone that acts through activation of soluble guanylyl cyclase (sGC). We hypothesized that increased smooth muscle tone limiting airflow in COPD could result from reduced sGC. Herein, we investigated the expression and downstream signalling of sGC in RB.

## Materials and methods

C57BL/6 mice were subjected to RB by restricting tracheal diameter by 50% using a nylon band. Animals were divided into the following groups: 1. Wild-type sham operated mice. 2. Wild-type mice subjected to tracheal banding (TB). 3. sGC-α1 -/- sham operated mice. 4. sGC-α1 -/- TB mice. 5. Wild-type sham operated mice treated with inhaled sGC inhibitor, (ODQ; 20mg/Kg). 6. Wild-type TB mice treated with inhaled sGC inhibitor, (ODQ; 20mg/Kg). 7. Wild-type sham operated mice treated with the sGC activator, BAY 58-2667(10µg/Kg; ip). 8. Wild-type TB mice treated with sGC activator, BAY 58-2667(10µg/Kg; ip).

## Results

Mice subjected to TB, exhibited a significant increase in BALF cellularity and protein content, consistent with the presence of acute inflammation. TB resulted in an increase in tissue elasticity and airway resistance and in a downward shift of the pressure-volume curve. TB reduced the expression of both α1 and β1 subunits of sGC, at mRNA and protein level in the lung. A pronounced inflammatory response was observed after ODQ administration or by knocking out the sGC α1 locus. Pharmacological activation of sGC, using BAY 58-2667, 30 min before or 24 hrs after TB, reversed the effects of RB in the lung.

## Conclusions

Our results indicate that sGC activation protects TB mice by reducing inflammation and improving lung mechanics and raise the possibility that sGC could potentially be used to ameliorate lung function in COPD patients.

